# Features generated for computational splice-site prediction correspond to functional elements

**DOI:** 10.1186/1471-2105-8-410

**Published:** 2007-10-24

**Authors:** Rezarta Islamaj Dogan, Lise Getoor, W John Wilbur, Stephen M Mount

**Affiliations:** 1 Computer Science Department, University of Maryland, College Park, MD 20742, USA; 2 National Center for Biotechnology Information, National Library of Medicine, National Institutes of Health, Bethesda, MD 20894, USA; 3 Department of Cell Biology and Molecular Genetics, University of Maryland, College Park, MD 20742, USA; 4 Center for Bioinformatics and Computational Biology, University of Maryland, College Park, MD 20742, USA

## Abstract

**Background:**

Accurate selection of splice sites during the splicing of precursors to messenger RNA requires both relatively well-characterized signals at the splice sites and auxiliary signals in the adjacent exons and introns. We previously described a feature generation algorithm (FGA) that is capable of achieving high classification accuracy on human 3' splice sites. In this paper, we extend the splice-site prediction to 5' splice sites and explore the generated features for biologically meaningful splicing signals.

**Results:**

We present examples from the observed features that correspond to known signals, both core signals (including the branch site and pyrimidine tract) and auxiliary signals (including GGG triplets and exon splicing enhancers). We present evidence that features identified by FGA include splicing signals not found by other methods.

**Conclusion:**

Our generated features capture known biological signals in the expected sequence interval flanking splice sites. The method can be easily applied to other species and to similar classification problems, such as tissue-specific regulatory elements, polyadenylation sites, promoters, etc.

## Background

The analysis of genome sequences in order to discover the location and structure of genes is an increasingly important task. However, a complete and accurate description of the gene structure on the basis of sequence alone remains a difficult problem [1]. In eukaryotic organisms, sequences known as *introns *are removed from precursors to mRNA, in the complex process of splicing. The boundaries between introns and exons are called *splice sites *and the identification of these positions poses a particular challenge. The adjacent nucleotides on intron boundaries comprise two different consensus sequences for the 5' (donor) site and 3' (acceptor) site. Position-specific scoring matrices can be compiled from thousands of annotated splice sites that reflect the contribution of each base at each position. Any given sequence can then be evaluated on the degree of agreement with the consensus matrix. However, similar sequences within introns and exons that fit the scoring matrices are observed at a very high frequency, and information at the 5' splice site, branch site, and 3' splice site is insufficient to accurately predict splicing outcomes [2]. These facts suggest that other factors must also play a role and help the complex of RNA and proteins identify real splice sites [3].

In many cases, the discrimination between splice sites and other sequences can be optimized using machine-learning methods. A machine-learning algorithm uses a set of known examples (the training set) and a set of characteristics or *features *describing the training set to construct a model of the data. The learned model is evaluated by testing its accuracy on a held-out test set. Different machine-learning algorithms, such as Markov models or neural networks, have been used to improve splice-site prediction [4]. GeneSplicer, described by Pertea et al. [5] and MaxEnt, described by Yeo and Burge [6], are examples of machine-learning algorithms applied to splice-site prediction. GeneSplicer uses Markov modelling techniques, in addition to Maximal Dependency Decomposition analysis, and MaxEnt uses a maximum entropy approach to rank and select "constraints" (features) for splice-site prediction.

An important input to any machine-learning algorithm is the choice of features describing the dataset. A challenge is how to determine the best set of features for the prediction task at hand. This is especially true for sequence data. One solution is to use automated feature-selection techniques that identify useful or informative features from a large collection of features.

Feature-selection techniques have been used extensively in machine-learning problems, and they have been receiving more attention in the computational biology community. For example, Liu and Wong used feature-selection methods in their prediction of translation-initiation sites [7]. Degroeve et al. [8,9] used feature subset selection, combined with support vector machines, to predict splice sites. Zhang et al. [10], employed a recursive feature-selection technique, based on support vector machines, to identify sequence information that distinguishes real exons from pseudo exons.

In earlier work, we developed a feature-generation algorithm (FGA) for sequence classification [11]. The algorithm used the four nucleotides of the DNA alphabet, {A, C, G and T}, and their positions in the sequence to construct descriptive features. FGA started with these basic features and built more-complex features in an iterative fashion. These features were: groups of consecutive nucleotides, groups of not-necessarily-adjacent nucleotides, and nucleotides or groups of nucleotides associated with particular positions or a range of relative positions in the sequence. Because the feature space explored was very large, FGA iteratively reduced the size of the feature set by eliminating features according to various feature-selection methods. Then, the final set of features that we obtained became input for the learning algorithm.

**Figure 1:**
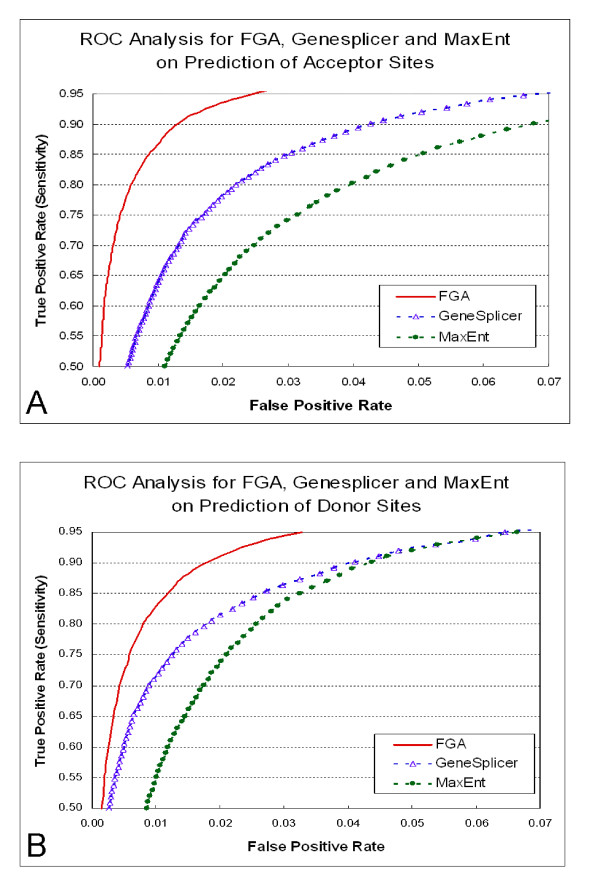
**Receiver Operating Curve Analysis for FGA, GeneSplicer and MaxEnt for Acceptor (A) and Donor (B) Splice-Site Prediction**. The true positive rate (TP/(TP+FN)) is plotted versus the false positive rate (FP/(FP+TN)). We show the sensitivity values ranging from 50% to 95%. When the score threshold for each method is adjusted such that 5% of the true sites are missed (sensitivity is 95%), for acceptor splice-site prediction, MaxEnt has recalled 10.48 % of the false sites, GeneSplicer 5.80% and FGA only 2.49%, and, for donor splice-site prediction, MaxEnt has recalled 6.61 % of the false sites, GeneSplicer 6.40% and FGA only 3.30%. These results are computed on the Human dataset of GeneSplicer team which contains 1,115 pre-mRNA sequences.

The learning algorithm that we used (C-Modified Least Squares, CMLS) is a max-margin classifier similar to support vector machines (Zhang and Oles, [12]). Relative to support vector machines, the CMLS algorithm exhibits a faster convergence, resulting in shorter training times. Our generated features, in combination with the CMLS classifier, resulted in two very effective splice-site prediction models for acceptor [11] and donor sites. We illustrate the performance of the FGA model for acceptor and donor splice-site prediction in Figure 1A and Figure 1B. Here we also include a comparison with the performances of GeneSplicer and MaxEnt splice-site prediction models. The FGA classifier has been made generally available as a webserver [13].

In this paper, we explore the knowledge-discovery power of our algorithm by taking a closer look at the generated features. We present examples of the observed feature groups and describe our efforts to detect biological signals that may be important for the splicing process. We find that the features generated for computational splice-site prediction include known functional elements, and we present evidence that these features provide previously unknown information about some aspects of these splicing signals.

## Results and discussion

### Sequences and splice-site neighborhood

For these experiments we considered canonical splice sites. We explored a splice-site neighborhood of 80 nucleotides upstream and 80 nucleotides downstream of the consensus AG or GT dinucleotides, with a total sequence length of 162 nucleotides. The sequence alphabet was composed of four different nucleotides A, C, G and T, and their individual positions were measured relative to the annotated splice site.

### Description of generated feature sets

Here we summarize the specific steps used to generate the composite feature sets used in our analysis. These features are significantly more complex than the features previously considered in the literature. The algorithm, FGA, is described formally in the Methods section and in [11]. To generate a composite feature set we need to specify an initial set of features, an appropriate construction method, and a fast feature-selection method. To prepare the initial sets of features, we started with the position-specific *k*-mer sets for *k *from *3 *to *6*. The numbers of potential features for these feature sets are, respectively, *10,240, 40,960, 163,840, and 655,360*. For each of these sets the *Information Gain *feature-selection method was used to select the top scoring *5,000 *features. These sets constituted our initial feature sets for the construction algorithm. As described in Methods, the feature-construction method expanded each of these sets by adding one position-specific nucleotide in an unconstrained position. After the construction step, we again used Information Gain to evaluate each of the features in the constructed set. Then we evaluated each feature according to a logistic scheme, taking into account the distance between the newly added nucleotide and the original feature, preferring features for which the distance was smaller. After the feature selection step, the top scoring *5,000 *features were selected. These sets constituted the input sets for the next iteration. We ran the algorithm and generated features up to, at most, 10 conjunct nucleotides in different positions in the composite feature sets. For each set of features we built a separate splice-site prediction model using the CMLS [12] classification algorithm. Table 1 summarizes the splice-site prediction performance for each of these feature sets. Some of these sets performed better than others, but in our analysis we explored all the sets for the purpose of knowledge discovery.

**Table 1:** Individual classification performances of FGA-generated feature sets for 3' (A-KmerX) and 5' (D-KmerX) splice sites.

A-3mer0	86.46						
A-3mer1	84.16	A-4mer0	84.92				
A-3mer2	77.01	A-4mer1	77.28	A-5mer0	80.60		
A-3mer3	69.42	A-4mer2	69.10	A-5mer1	69.20	A-6mer0	68.64
A-3mer4	63.30	A-4mer3	63.11	A-5mer2	62.74	A-6mer1	61.72
A-3mer5	56.84	A-4mer4	56.66	A-5mer3	56.25	A-6mer2	54.65
A-3mer6	49.50	A-4mer5	49.23	A-5mer4	49.08	A-6mer3	47.19
A-3mer7	41.22	A-4mer6	41.02	A-5mer5	40.51	A-6mer4	39.62

D-3mer0	86.79						
D-3mer1	83.45	D-4mer0	85.21				
D-3mer2	80.31	D-4mer1	81.14	D-5mer0	83.64		
D-3mer3	70.08	D-4mer2	70.47	D-5mer1	77.20	D-6mer0	75.03
D-3mer4	56.06	D-4mer3	55.38	D-5mer2	57.42	D-6mer1	66.68
D-3mer5	42.97	D-4mer4	44.77	D-5mer3	38.09	D-6mer2	43.31

FGA-generated feature sets for splice sites and their individual performances at splice-site prediction. Each value reported is an average precision (positive predictive value, TP/(TP+FP)) over 11 values of recall (sensitivity, TP/(TP+FN)), 0%, 10%, 20% ... and 100%, and is the result of a three-fold cross validation. All the features in these features sets extend along the whole splice-site neighbourhood [-82, 80] that we study.

In what follows, we use the shorthand notation *S*-*kMERn*[*p*_1_, *p*_2_] to describe the composite feature subsets that we studied. In this notation, *S *∈ {*A*, *D*} stands for acceptor (*A*) or donor (*D*) splice sites, *kMER *stands for the number of consecutive position-specific nucleotide features in the initial set, *n *is the number of additional conjuncts and [*p*1, *p*2] denotes the interval from position *p*1 to position *p*2 in the sequence. For example, *A*-3*mer*3[20,40] is a subset of acceptor splice-site features. These features were generated from the initial set of position-specific 3-mer features and were obtained after three FGA iterations, adding each time a new nucleotide in an unconstrained position within the specified interval. The sequence positions associated with each of the features in this subset were from the coding region 20 to 40 nucleotides downstream the acceptor splice site.

Following with our definitions, we say that two composite features match if they share the same nucleotide pattern, starting at different positions. For example, let 4*mer*[1,10] = {*a*_1 _*g*_2 _*c*_3 _*t*_4_, *a*_6 _*g*_7 _*c*_8 _*t*_9_} be the subset of composite 4-mer features from the interval [1,10], where *a*_1 _denotes nucleotide *a *at the first sequence position. In this case, the features *a*_1 _*g*_2 _*c*_3 _*t*_4 _and *a*_6 _*g*_7 _*c*_8 _*t*_9_, are two *matching composite features*. A composite feature subset may contain several matching features that differ only in the starting position within the specified interval. We represent a set of such occurrences with an *interval-feature pattern*, e.g. *a*_*i *_*g*_*i*+1 _*c*_*i*+2 _*t*_*i*+3_. An interval-feature pattern is the nucleotide pattern shared among the matching composite features and the number of interval occurrences of a feature pattern is the number of matching composite features it represents. We use the notation *S*-*kMERn*[*p*_1_, *p*_2_]* to denote the set of all interval- feature patterns for the subset *S*-*kMERn*[*p*_1_, *p*_2_]. For the above example, given the set of features 4*mer*[1,10] = {*a*_1 _*g*_2 _*c*_3 _*t*_4_, *a*_6 _*g*_7 _*c*_8 _*t*_9_}, the set of interval-feature patterns is 4*mer*[1,10]* = {*a*_*i *_*g*_*i*+1 _*c*_*i*+2 _*t*_*i*+3_}. The number of occurrences for the pattern *a*_*i *_*g*_*i*+1 _*c*_*i*+2 _*t*_*i*+3 _in the given feature set is two.

In our analysis, features were ranked according to the weight assigned to them by the classification algorithm. We used the WebLogo program [14] to draw frequency plots. We plotted histograms and used basic k-means clustering algorithms and edit-distance measures to cluster the features into groups. Here we list some of our findings and illustrate them with our features.

### Knowledge discovery: generated features capture biological signals

What kinds of biological signals do these generated features capture? Examples of positive signals that we might expect to find in a typical pre-mRNA include the branch site, the pyrimidine-rich region close to the acceptor splice site, splice-site consensus signals themselves, and exonic splicing enhancers. In addition, it is likely that sequence elements associated with the coding sequence were present among our features. However, we found that FGA performed quite well (the 11-point average precisions for acceptor and donor splice sites were, respectively, 83.33% and 64.52%) on the recognition of splice sites flanked by non-coding exons (data not shown).

### The Branch Site interval

The mammalian branch-site signal is difficult to describe because it is degenerate and shows very low levels of purifying selection [15]. In order to investigate the branch-point signal, we examined composite features of 6 nucleotides that start in the interval from 40 to 20 nucleotides upstream from the acceptor splice site (and therefore extend from -40 to -15). Our current feature set for this purpose was *A*-3*mer*3[-40,-20]. The subset contained 346 selected features.

Table 2 shows the top-scoring 20 features in their exact position with respect to the annotated acceptor site, which is found 15 nucleotides downstream of the interval shown. Each feature is listed, ranked by the weight assigned by the CMLS classification algorithm. A large number of positional features in this feature set captured the branch-point signal. In fact, of the 30 features that had weights above 0.1 in this set, all but 5 contained either CTRA or at least five pyrimidines. In absolute numbers, 97 individual features of this set matched the branch-point consensus TNCTRAC [16] and 158 features were pyrimidine-rich. The rest of the features were assigned negative weights. The negatively weighted features comprised a G-rich signal mostly. Of those, 44 features matched the pattern AGG and the others were A-rich (see supplemental data, additional file 1).

**Table 2:** Top scoring features in branch site interval

**FGA A-3mer3 [-40,-20] features**	**Weight**
------------ctgacc-------	0.1800
-----------ctgacc--------	0.1678
----------------ctgacc---	0.1488
----------ctgacc---------	0.1453
-------------cctgac------	0.1417
---------------cctgac----	0.1382
----------------tgaccc---	0.1371
--------ctgacc-----------	0.1370
-----------------cctgac--	0.1368
------ctgacc-------------	0.1359
--------------ctgacc-----	0.1358
-------------------tctctc	0.1303
------------------ccttct-	0.1283
-------------------cttttc	0.1281
------------------cttttt-	0.1281
-------------ctcacc------	0.1254
-----------ctcacc--------	0.1219
---------------ctgact----	0.1206
-----------cctgac--------	0.1202
-------------------tccctc	0.1200

The 20 top-scoring *A *-3*mer*3 [-40,-20] features (i.e. composite features that start in the interval between -40 and -25 derived using FGA from a seed of trimers) are all related to either the branch-site consensus or the pyrimidine tract.

Table 3 illustrates a subset of *A-*3*mer*3[-40,-20]* interval-feature patterns. Each listed pattern represents at least five matching composite features, differing only in the starting position in this interval. The number of interval occurrences is also given and an average weight is computed for each interval-feature pattern from the individual CMLS weights assigned to the distinct matching composite features during training. We grouped these patterns into three categories: 1) nine interval-feature patterns matching the branch-site consensus, 2) two pyrimidine-rich interval-feature patterns, and 3) two negatively weighted purine-rich interval-feature patterns.

**Table 3:** Identified interval-feature patterns in the branch-point interval

A-3mer3 [-40,-20]*	**Interval occurrences**	**Average Weight**	**Total occurrences**	**Total Range**
--cctgac--	10	0.096	13	[-34,-16]
---ctgacc-	9	0.131	12	[-33,-16]
---ctgact-	8	0.082	11	[-32,-16]
-ccctga---	7	0.083	7	[-32,-19]
--gctgac--	7	0.083	8	[-34,-18]
--tctgac--	7	0.083	8	[-32,-18]
----tgaccc	6	0.089	9	[-32,-16]
--actgac--	5	0.059	6	[-33,-13]
---ctgatg-	5	0.068	7	[-36, 18]
				
-cccctc---	7	0.065	24	[-35, 0]
---cctctc-	5	0.049	22	[-36, 0]
				
--gggagg--	6	-0.041	23	[-34, 14]
--aaaaaa--	5	-0.028	84	[-50, 80]

The first column shows the interval-feature patterns in the branch-point interval [-40,-20]. The second column shows the number of individual occurrences for each pattern in different positions within the specified interval. The average assigned weight is given in the third column. For comparison we include the total number of occurrences for this pattern in the complete neighbourhood ([-82, 80]) (forth column), and in the last column we show the narrowed range interval that comprises the total occurrences for each pattern.

**Figure 2:**
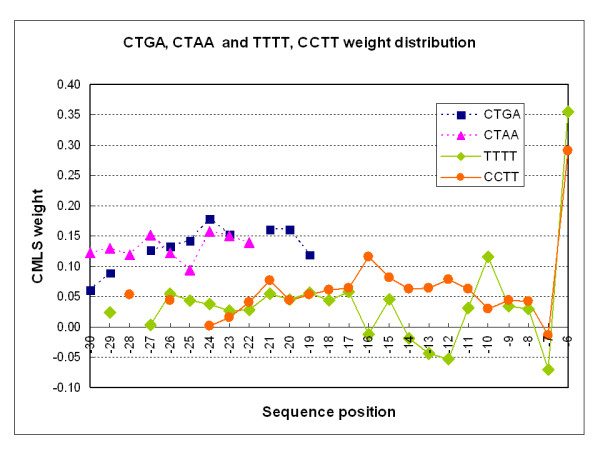
**Weight distribution comparison for pairs of tetramers CTGA, CTAA and TTTT, CCTT**. The distribution of CMLS weights for four tetramers from *A*-3*mer*1 [-60,-5] is shown graphically. Note that the distributions of scores for CTGA and CTAA are similar and sharply focused around the peak that would place the branch A at position -24. Note that the distributions of TTTT and CCTT corresponds to the well-known pyrimidine tract with the additional information that C is preferred to T at positions -15 through -11, where a peak of scores for CCTT coincides with a group of negative values for TTTT. There are no occurrences of these four hexamers in this feature set upstream of the region shown.

Table 4 lists all the position-specific occurrences of GCTGAC in the [-80, -1] interval. These features matched the branch-site consensus and they were assigned positive weights by the classification algorithm. The distribution of scores for this one hexamer suggests a preferred location for the branch site A at -30 to -20. Many independent observations with related features (*e.g*. CTAAC) indicated a similar region. For example, in Figure 2, we present a comparison of four tetramer features present in the *A*-3*mer*1[-60,-5] set. It is apparent from the distribution of these features that positions -27 through -16 are preferred for the branch site A. This observation agrees well with experimental results [17].

**Table 4:** Individual position-specific GCTGAC features

**Features in exact position wrt AG consensus**	**Weight**
-----------gctgac---------------------AG	0.114
----------------gctgac----------------AG	0.114
---------------gctgac-----------------AG	0.105
----------gctgac----------------------AG	0.082
------------gctgac--------------------AG	0.077
------gctgac--------------------------AG	0.074
---------gctgac-----------------------AG	0.068
-------------gctgac-------------------AG	0.062

A summary of position-specific GCTGAC features and their respective weight assigned by the CMLS classifier from the *A *-3*mer*3 [-80.-1] feature set.

**Figure 3:**
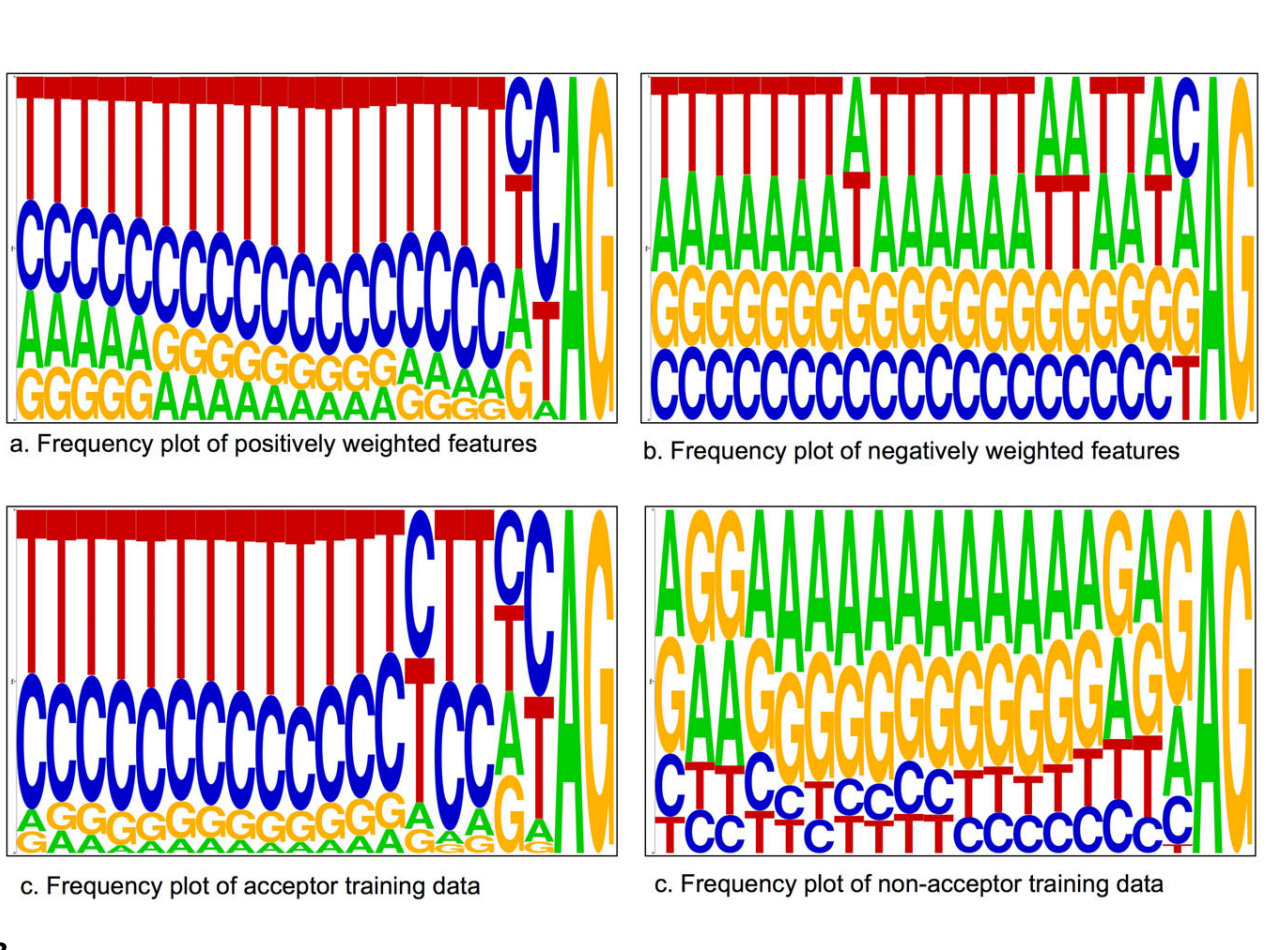
**The acceptor splice-site (pyrimidine-tract) interval**. Frequency plot sequence logos for the positively and negatively weighted features in the pyrimidine-tract interval, *A*-5*mer*1 [-20,-1], (Figure 3a and Figure 3b), compared with frequency distribution of the training acceptor and non-acceptor sequences in the same interval (Figure 3c and Figure 3d). The positive features frequency plot corresponds to the acceptor splice-site consensus, which is also illustrated with the true acceptor sequences frequency plot. The negative features frequency plot reveals an AG-rich element.

### The acceptor splice-site (pyrimidine-tract) interval

Also shown in Figure 2 is the distribution of TTTT and CCTT in this interval. Note that this distribution is broader than the distribution of branch-site tetramers. In addition, there is a region (-16 to -12) where the scores assigned to TTTT become negative and tetramers containing C have maximal scores. Similar peaks are observed for CTTT, TCTT, TTCT and TTTC (see supplemental data, additional file 2).

In order to further investigate the characteristics of the upstream region close to the acceptor splice site, we also examined the feature set *A*-5*mer*[-20,-1] There were more than 2,000 selected features in this subset. We note that a large number of features were selected in this set, indicating stronger potential signals close to the splice site. Based on the weight assigned by the CMLS algorithm, we divided these features into two groups; positively weighted features and negatively weighted ones. In Figure 3, we used the WebLogo program to draw a frequency plot of the two groups of features. The annotated acceptor site is shown in the figure with the consensus dinucleotide AG.

One interpretation from these plots is that the generated features are capturing the pyrimidine tract, and that they are scanning along the sequence for the exact AG dinucleotide consensus where the true acceptor site is located. The difference between the two frequency plots for positively and negatively weighted features is striking. Figure 3a shows that the presence of the CT-rich feature is very important in this interval and Figure 3b shows that the presence of an AG-rich element is an indicator of a non-splice sequence. The frequency plot for the positively weighted features (Fig. 3a) is very similar to the acceptor splice-site consensus itself. However, our features do not simply reflect the nucleotide frequencies seen at true sites. Figures 3c and 3d show the frequency distribution of the true acceptor sequences and non-acceptor sequences in the training dataset. The frequency distribution of the non-acceptor sequences in our dataset in the pyrimidine-tract interval (Fig. 3d) is different from that of the negatively weighted features in the *A***-**5*mer*[-20,-1] feature set (Fig. 3b). In other words, our features were better than frequency data alone at discriminating true splice sites. To illustrate this difference, we used the frequency distribution matrices of these data to discriminate the true splice sites, achieving an 11ptAvg precision of 40.1%. On the other hand, when we trained a CMLS classifier on the FGA feature set, it achieved an 11ptAvg precision of 80.6% for the same task.

**Figure 4:**
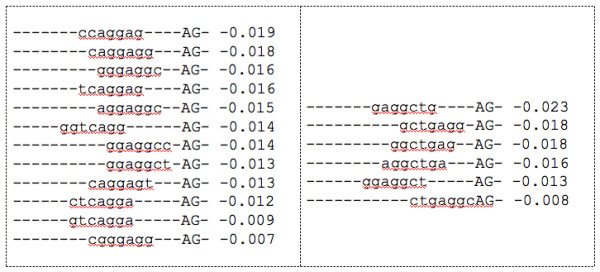
**Clusters of negative features of the pyrimidine-tract interval**. Examples of the individual features for two clusters of features and the assigned CMLS weight for each feature from the feature set *A*-6*mer*1 [-20,-1]. The presence of the AG dinucleotide upstream the annotated 3' splice site, in the pyrimidine-tract interval is not preferred. All these features have negative weights.

Exploring the pyrimidine-tract interval further, we selected another feature set, which was characterized by composite positional features containing 7 nucleotides in different positions, *A*-6*mer*1[-20,-1]. We made a list of the features, and we identified clusters of similar features, using the *k*-means clustering algorithm with the edit-distance similarity measure. Figure 4 shows some examples and samples of the features in each group.

### GGG motifs near the 5' slice site

**Figure 5:**
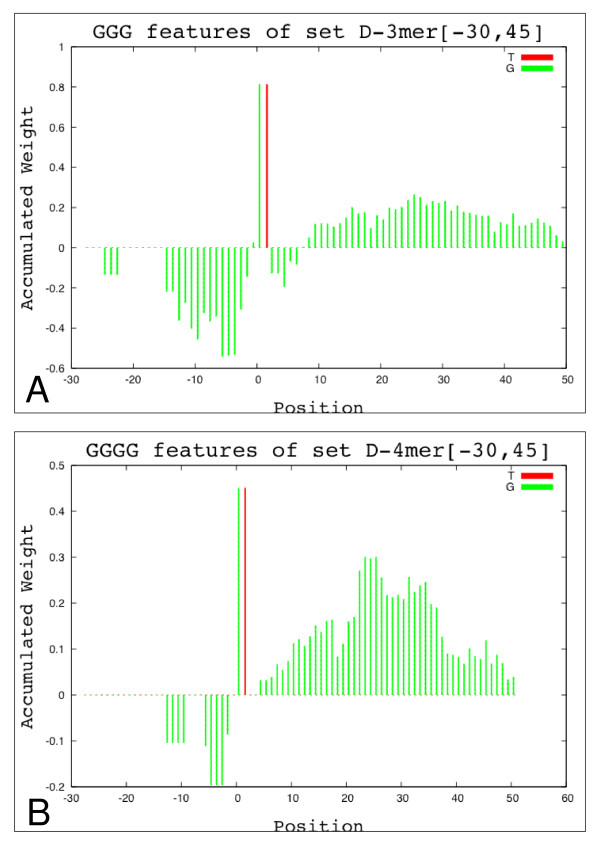
**G-rich features in the donor-site interval**. Weighted histogram for all the GGG (A) and GGGG (B) features in the donor-site interval selected from the feature set *D*-3*mer*1 [-30,-45] (A) and *D*-4*mer*1 [-30,-45] (B). These features are not preferred upstream the donor site, but they are encouraged on the downstream region.

In order to investigate the characteristics of introns near the 5' splice site, we explored the intron downstream of the 5' splice site, using a number of parameters. In each case, GGG and GGGG motifs were common. For example, the *D*-3*mer*[6,64] set included 54 positively ranked occurrences of GGG and 4 negatively ranked occurrences. A plot of scores versus position for GGG and GGGG is provided in Figure 5A and Figure 5B, showing that this motif scores positively in the intron downstream of 5' splice sites but negatively in the flanking exon. GGG likewise dominates *D*-3*mer*[-80,-40]. A number of papers have reported a role for GGG and GGGG motifs in splicing [18-20]. Recognition of these motifs has been attributed to the U1 snRNP [21] and hnRNP H [19].

### The donor splice-site interval

**Figure 6:**
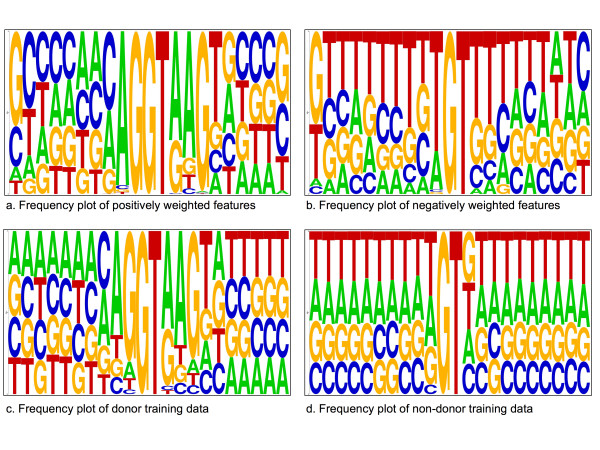
**The donor splice-site interval**. Frequency plot sequence logos for the positively and negatively weighted features in the donor-site interval, *D *-6*mer*[10,10] (Figure 6a and Figure 6b), compared with frequency distribution of the training donor and non-donor sequences in the same interval (Figure 6c and Figure 6d). The positively weighted features capture the donor-site consensus ([A|C]AGGT [A|G]AGT.

Next, we investigate the characteristics of the donor splice site. Sample clusters, similar to those created for the acceptor site, are shown in Figure 6. The first two sequence logos, Figure 6a and Figure 6b, show the frequency plot of the positively and negatively weighted groups of features for the set *D*-6*mer*[-10,10]. The donor splice-site consensus sequence is MAGGTRAGT (where M is A or C and R is A or G). The next two plots, Figure 6c and Figure 6d, show the frequency plot for the same interval based on the true donor and non-donor sequences in the training dataset. Once again, the sequence logo of the positively weighted features resembles the logo of the nucleotide frequency of the positive data, but important differences are apparent, especially at positions on the periphery of the region shown.

### Exon Splicing Enhancers (ESEs) and Exon Splicing Suppressors (ESSs)

We also compared our generated features to published work on Exonic Splicing Enhancers (ESEs) and Exonic Splicing Silencers (ESSs). ESEs and ESSs are short oligonucleotide sequences located in the exonic region that affect splicing. The presence of ESE sequences in the exonic region results in the enhancement of the recognition of the nearby splice sites. The presence of the ESS sequences, on the other hand, suppresses nearby splicing events. These regulatory signals have been studied experimentally (reviewed in [22]) and computational methods have been built to find them [23-28].

We considered the set of distinct hexamers in the flanking exon interval, for both acceptor and donor by computing interval features of the region of the sequence downstream from the annotated splice site for acceptor and upstream for donor. We divided this set of interval features into positively and negatively weighted sets. We compared these sets of hexamers (see supplemental data, additional file 3) with a list of experimentally identified ESE's and ESS's of mammalian and viral RNA [22]. There are 61 experimentally determined ESE sequences listed by Zheng [22], including some that are identical but have different sources. The set of hexamers identified from our method produced an overlap for 54 ESE sequences comprising 641 nucleotides, out of 738, yielding a coverage of 87%. Twenty-eight of these sequences were perfectly identified by the hexamers covering all the nucleotides. The ESS sequences were not recognized as well as the ESE ones. We provide these results as supplement data (see supplemental data, additional file 4).

Rescue-ESE [24], Fas-ESS [25] and ESR [26] are computational methods that are specifically tailored to identifying exonic signals that impact a splicing event. Rescue-ESE identified candidate exonic splicing enhancers in vertebrate exons based on their statistical features. This method identified a set of 238 hexamers, which we refer to as RescueESE. Fas-ESS started with a set of experimentally identified exonic splicing silencer sequences of length 10. It computationally derived a set of 176 hexamers which we refer to as FasESS. ESR identified exonic splicing regulator sequences based on conservation of synonymous nucleotides. This set contains 285 hexamers, which were not necessarily divided into enhancer and silencer categories. We refer to this set as AstESR. An additional method (Zhang and Chasin, [29,30]) compared *bona fide *exons with pseudo-exons in order to identify putative ESEs (PESEs) and putative ESSs (PESSs). The PESE set contains 2060 octamers and the PESS set contains 1018 octamers. There were 1701 unique hexamers in the PESE set, which we refer to as ChPESE, and there were 924 unique hexamers in the PESS set, which we refer to as ChPESS.

In order to be able to compare the FGA-generated features with the ESE hexamers identified by these methods, we looked at the different FGA sets of features that contained six consecutive position-specific nucleotides and were associated with the exonic regions. We looked at the feature sets generated for both acceptor and donor splice sites. We selected the features that belonged to the sequence interval 80 nucleotides downstream of annotated acceptor splice sites and 80 nucleotides upstream of annotated donor sites (bearing in mind that these intervals can contain some contribution from the adjacent intron that lies beyond the exon). Because FGA features were position-specific, for each set we computed the interval-feature patterns, thus obtaining a list of hexamers found in the exonic regions. We divided the features into positively weighted and negatively weighted sets denoted as *S*-*kMERn*[*p*_1_, *p*_2_]+ and *S*-*kMERn*[*p*_1_, *p*_2_]-, where *S *∈ {*A*, *D*} stands for acceptor and donor features respectively.

We computed the overlap between each FGA-generated set of hexamers and each of the four published sets of exonic regulatory sequences (see supplemental data, additional file 5). We present the overlap for each pair of sets and the corresponding p-values in Table 5. The p-value shows the probability that a randomly selected set of hexamers, containing as many hexamer features as found by the FGA algorithm, has an overlap equal to or greater than the value given in the *Overlap *column in Table 5; this probability is calculated from the hypergeometric distribution. In Table 5, we have highlighted all the p-values less than 0.01 or greater than 0.99, indicating the significant relationship between the feature sets. All of these other sets have significant overlaps with our features, but the most significant are with ChPESE and ChPESS sets, perhaps because they were generated using methods similar to ours.

**Table 5:** FGA-generated feature set show significant overlap with ESE and ESS regulator signal sets.

***FGAset***	***size***	***AstESR (285)***		***RescueESE (238)***		***ChPESE (1701)***		***FasESS (176)***		***ChPESS (924)***	
		***Overlap, P-value***		***Overlap, P-value***		***Overlap, P-value***		***Overlap, P-value***		***Overlap, P-value***	
A-3mer3 [1,80]	313	34	**0.00514**	24	0.09415	175	**2.09e-06**	10	0.877	73	0.5407
A-3mer3 [1,80]+	177	28	**0.00003**	24	**0.00007**	130	**1.42e-18**	1	**0.999**	8	*
A-3mer3 [1,80]-	136	6	0.92089	0	*	43	0.9939	9	0.129	59	**3.19e-08**
											
A-4mer2 [1,80]	317	35	**0.00347**	26	0.04319	177	**1.96e-06**	10	0.887	72	0.6423
A-4mer2 [1,80]+	179	29	**0.00001**	25	**0.00003**	129	**2.74e-17**	1	**0.999**	9	*
A-4mer2 [1,80]-	138	6	0.92714	1	**0.99999**	46	0.9819	9	0.137	57	**4.22e-07**
											
A-5mer1 [1,80]	342	35	0.01147	27	0.05920	278	**1.06e-08**	12	0.812	70	0.9300
A-5mer1 [1,80]+	187	29	**0.00003**	25	**0.00006**	134	**1.40e-17**	3	**0.999**	9	*
A-5mer1 [1,80]-	155	6	0.96496	2	**0.99915**	59	0.8352	9	0.221	54	**0.000257**
											
A-6mer [1,80]	465	54	**0.00006**	27	0.53401	278	**1.06e-08**	17	0.799	91	**0.9993**
A-6mer [1,80]+	263	38	**0.00001**	25	**0.00899**	165	**6.61e-13**	7	0.943	19	*
A-6mer [1,80]-	202	16	0.32994	2	**0.99984**	76	0.8907	10	0.368	64	**0.001374**
											
D-5mer1 [-80,-1]	64	10	0.01195	32	**1.32e-23**	60	**5.59e-19**	1	0.941	4	**0.9999**
D-5mer1 [-80,-1]+	56	9	0.01403	30	**2.47e-23**	52	**4.27e-16**	0	*	4	**0.9995**
											
D-6mer [-80,-1]	1052	126	**1.44e-12**	112	**1.81e-13**	613	**3.73e-37**	26	**0.999**	183	**0.9999**
D-6mer [-80,-1]+	701	93	**2.28e-11**	109	**6.16e-28**	482	**1.02e-57**	6	**0.999**	63	*
D-6mer [-80,-1]-	271	20	0.42504	1	**0.99999**	90	0.9985	19	0.022	106	**1.54e-10**

* p-value is very close to 1.

The number of shared features between the FGA generated sets of hexamers and the exon regulator hexamer sets and the p-value stating the probability of having this overlap or a greater overlap by chance. We highlight the highly statistically significant probabilities. The set *D *-3*mer*3 [-80,-1] did not contain position specific hexamers and the set *D *-4*mer*2 [-80,-1] contained only 3 position specific hexamers, two of which overlapped with RescueESE set.

**Figure 7:**
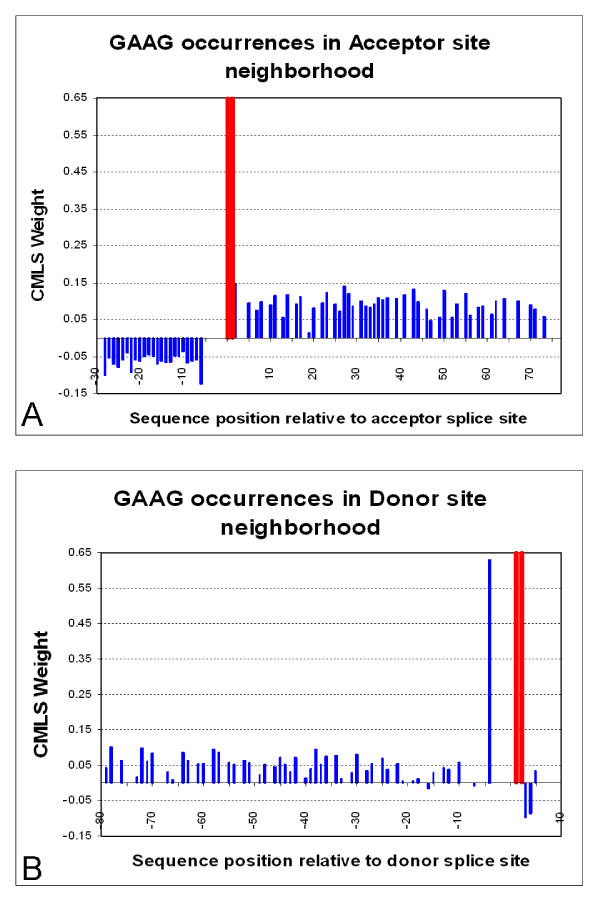
**The weight distribution of the ESE motif GAAG in the acceptor (A) and donor (B) splice-site neighborhood**. The x-axis shows the acceptor splice-site neighborhood interval. The consensus dinucleotide AG location is marked with the red bars (positions -1 and -2) in Figure 7A. The consensus dinucleotide GT location is marked with the red bars (positions 1 and 2), in Figure 7B. For every occurrence of the feature GAAG in the set *A-*4*mer *[-80,80], we draw a bar corresponding in height to its CMLS assigned weight. This feature has a negative weight when it is positioned in the intron region, but a positive weight downstream the splice site. For the donor site, we notice its exceptionally high weight at position -4. One possible reason may be the reflection of the donor-site consensus signal.

In order to address possible positional preferences [31] for ESE elements we examined the distribution of short motifs corresponding to ESEs among our features. We observed a clear preference for exon sequences, but did not find a strong preference for a particular interval or position. For example, the GAAG tetramer is weighted positively throughout the exonic region, as illustrated in Figure 7A and Figure 7B. This signal was found in almost every position in the 80 nucleotide region and the weights of the respective features were very similar, so we cannot specify a region or interval of preference. The one exception was the immediate neighborhood of the donor site (position -4), which reflects splice-site consensus rather than exonic splicing enhancer signal. In contrast, GAAG was a negatively weighted feature in the intronic region.

We next asked whether those hexamers present in our set but not others have predictive value. As described above, many experimentally determined exonic enhancers (as reviewed by Zheng [22]) overlapped our features. While this was true of the other sets as well, even when those previously described motifs were excluded, our features still accounted for some observations (see supplemental data, additional file 4). Interestingly, many of these were examples of the A/C-rich motifs: CACACA, GCCCAA, TCAACA, CATTCA and CCTACA. Such A/C-rich elements have been described before [32] but have not been extensively characterized.

## Conclusion

We previously showed that our FGA algorithm could be used to build accurate sequence classifiers [11]. Here we have shown that the features generated by our algorithm for the purpose of discriminating between true and false splice sites correspond to functional splicing signals. Generated features included known features such as the branch-site consensus, acceptor splice-site consensus, pyrimidine tracts, coding potential and exon splicing regulator signals. The ability of FGA to accurately extract the branch-site signal (Tables 2, 3, 4) is especially noteworthy in view of the elusive nature of this signal [15]. Furthermore, the generated features provided information about the preferred location and sequence of these features, as illustrated by the distribution of branch-site and pyrimidine-tract features. However, we note that because FGA does not produce features to capture particular events such as AG di-nucleotide exclusion zones [33], it was not able to extract contingent signals such as distant branch sites coupled to them.

In addition, novel aspects of splicing signals could also be inferred from this method. We point to two examples. One is the co-occurrence of a peak of CCTT scores with a group of negative CMLS weights for TTTT at position -11 in the acceptor region. We believe that this may be a real, and previously unappreciated, aspect of the pyrimidine-tract signal. This signal is recognized by the large subunit of U2AF (and by PUF60; [34]). We note that in-vitro selection experiments [35] found a marked preference for a CC dinucleotide in the case of U2AF but not PTB or Sxl. Thus, although U2AF will bind oligoU, there are other proteins that will do so and these are generally splicing repressors. Our observed features were consistent with the possibility that positions -12 and -11 may be an especially important region for discriminating between positive factors and negative factors that bind to similar sequence elements. This subtlety was revealed by our features despite the fact that it was not apparent from raw nucleotide-frequency data (Fig. 3). In a second example, even though our ESE hexamer features showed a statistically significant overlap with those obtained by other computational methods (Table 5), there were examples obtained by ours but not other methods, including a number of ESE motifs that corresponded to experimentally determined exonic splicing enhancers.

Finally, this method can be easily applied to other species and to similar classification problems for the discovery of species-specific regulatory elements. We have made our features available online ([13].

## Methods

### Dataset

We have used a dataset of 4,000 human RefSeq pre-mRNA sequences to generate features and train our classifiers. A splice-site sequence in our training data is a subsequence consisting of 80 nucleotides upstream from the annotated splice site and 80 nucleotides downstream [80+AG/GT+80]. We counted the borders of all the introns within protein-coding regions whose acceptor and donor sites followed the AG and GT dinucleotide consensus. In order to construct negative examples for the training datasets, we selected random AG-pair or GT-pair locations that were not annotated splice sites and collected the subsequences as we did for the true sites. Our acceptor site training set consisted of 20,996 positive instances and 200,000 negative instances. Our donor-site training set consisted of 20,761 positive instances and 200,000 negative instances. We did not remove the sequences found within the regions identified by RepeatMasker. When we ran RepeatMasker on our training sets of sequences, we marked those sequences which had at least 20% of their nucleotides "masked" and the masking included the splice-site location. They constituted 36 of our positive and 67,571 of our negative instances. Our experiments revealed that the FGA performance was not affected by the repeated elements and the changes in the results when we did not include the repeated sequences in our training data were not significant. Therefore, all training was developed on the original training sequences, using a three-fold cross-validation scheme.

### Splice-site prediction model and performance evaluation

Our feature generation algorithm [11] uses the pre-mRNA sequence properties to construct and select useful features for splice-site prediction. Feature generation starts with an initial feature set. Then, the algorithm iteratively calls a feature-construction method to expand the current feature set, and it calls a feature-selection method to identify the useful features for the prediction task. After a specified number of iterations the algorithm produces an output feature set. The final set of features is then used as input to the learning algorithm for the sequence classification task.

We have used these features with a least-squares classifier algorithm, CMLS [12]. When compared to AdaBoost, Support Vector Machines, Naïve Bayes and Maximum Entropy, this was the classifier that consistently gave the best performance. CMLS is a linear classifier with a performance similar to linear support vector machines, but with a mach faster convergence and therefore a shorter training time. When the classifier is trained, each of the input features is assigned a weight. These weights define the hyperplane, the decision boundary that optimizes the performance. Then, each given sequence, is assigned a score by adding the weights of each feature that is present in the sequence.

We evaluated the performance of our model using 11-point average precision (11ptAvg Precision) and Receiver Operating Curve (ROC) analysis. For any sensitivity ratio, TP/(TP+FN), we calculate the precision at the threshold which achieves that ratio. Precision, TP/(TP+FP), measures the proportion of the sequences scoring above the threshold that are true splice sites. The 11ptAvg is the average of precisions estimated at these sensitivity values 0%, 10%, 20%, ..., 100%. We also draw the ROC curve, which is the graphical representation of the sensitivity (on the y-axis) versus false positive rate (on the x-axis). False positive rate, FP/(TN+FP), is the value we wish to minimize and the ROC graph shows the tradeoff between sensitivity and false positive rate.

### Feature types and construction procedures

The composite features we generated for splice-site prediction capture compositional and positional properties of sequences. In our general FGA technique, we distinguished different types and we defined a construction algorithm for each type. In the experiments described in this paper, we used positional composite features, which we define as follows:

*Position-specific nucleotides *are basic features that represent the nucleotides at each of the positions *i *in the sequence. These features capture the nucleotide-position preference in the sequence; therefore they are very commonly used in DNA sequence-classification analysis. As an example, assume our feature set is F = { *a*_1_, *c*_1_, ..., *g*_*n*_, *t*_*n*_}, where *a*_1 _denotes nucleotide *a *at the first sequence position. Our sequences have a length *n *of *162 *nucleotides; therefore our position-specific set of single nucleotides contains *648 *features. We use this initial feature set to construct complex position-specific features.

### Position-specific k-mer features

The *position-specific k-mer *features capture the correlations between k-adjacent nucleotides and their respective positions. At each position *i *in the sequence these features represent the substring appearing at positions *i*, *i *+ 1, ..., *i *+ *k *- 1.

#### Construction Method

Given an initial set of position-specific k-mer features, this construction method expands them to a set of position-specific (*k *+ 1)-mers by appending another nucleotide to each position-specific k-mer. Now, if our initial set is F_*intial *_= {*a*_1 _*g*_2_}, we can extend it to the set F_*constructed *_= {*a*_1 _*g*_2 _*a*_3_, *a*_1 _*g*_2 _*c*_3_, *a*_1 _*g*_2 _*g*_3_, *a*_1 _*g*_2 _*t*_3_}.

### Composite positional features

The *composite positional *features consist of a conjunction of *n *nucleotides in *n *different positions co-occurring in the sequence. In the simplest case, this type of feature set consists of position-specific single nucleotides. While the position-specific k-mers capture only the correlations among nearby positions, the composite positional features intend to capture the correlations between different nucleotides in non-consecutive positions in the sequence. We construct these complex features from conjunctions of position-specific features. The dimensionality of this kind of feature is inherently high. If the number of conjuncts is k, we have a total of (nk)
 MathType@MTEF@5@5@+=feaafiart1ev1aaatCvAUfKttLearuWrP9MDH5MBPbIqV92AaeXatLxBI9gBaebbnrfifHhDYfgasaacH8akY=wiFfYdH8Gipec8Eeeu0xXdbba9frFj0=OqFfea0dXdd9vqai=hGuQ8kuc9pgc9s8qqaq=dirpe0xb9q8qiLsFr0=vr0=vr0dc8meaabaqaciaacaGaaeqabaqabeGadaaakeaalmaabmaajugqbeaafaqabeGabaaabaGaemOBa4gabaGaem4AaSgaaaGccaGLOaGaayzkaaaaaa@31CA@ × 4^*k *^such features for a sequence of length *n*.

#### Construction Method

Given the set of *k*-conjuncts, this construction method selects from the set of basic features to add another position-specific nucleotide in an unconstrained position. In this manner we construct the set of (*k *+ 1)-conjuncts. Now, if our initial set is F_*initial *_= {*a*_1 _*g*_2_}, we can extend it to the level *2 *set of position-specific base combinations F_*constructed *_= {*a*_1 _*g*_2 _^ *a*_3_, *a*_1 _*g*_2 _^ *c*_3_, ..., *a*_1 _*g*_2 _^ *t*_*n*_}. Incrementally, in this manner we can construct higher levels.

### Feature Selection

Feature-selection methods reduce the set of features by keeping only the useful features for the task at hand. The problem of selecting useful features has been the focus of extensive research and many approaches have been proposed [36-39].

We considered several approaches for initial pruning of features of different types during the generation stage. In our data, we found that the Information Gain feature-selection method performed best for selecting composite positional features and we calculated the value for each of the features according to the following formula:

IG(f)=−H(C)+p(f)H(C/f)+p(f¯)H(C/f¯),
 MathType@MTEF@5@5@+=feaafiart1ev1aaatCvAUfKttLearuWrP9MDH5MBPbIqV92AaeXatLxBI9gBaebbnrfifHhDYfgasaacH8akY=wiFfYdH8Gipec8Eeeu0xXdbba9frFj0=OqFfea0dXdd9vqai=hGuQ8kuc9pgc9s8qqaq=dirpe0xb9q8qiLsFr0=vr0=vr0dc8meaabaqaciaacaGaaeqabaqabeGadaaakeaacqWGjbqscqWGhbWrcqGGOaakcqWGMbGzcqGGPaqkcqGH9aqpcqGHsislcqWGibasdaqadaqaaiabdoeadbGaayjkaiaawMcaaiabgUcaRiabdchaWnaabmaabaGaemOzaygacaGLOaGaayzkaaGaemisaG0aaeWaaeaadaWcgaqaaiabdoeadbqaaiabdAgaMbaaaiaawIcacaGLPaaacqGHRaWkcqWGWbaCdaqadaqaamaanaaabaGaemOzaygaaaGaayjkaiaawMcaaiabdIeainaabmaabaWaaSGbaeaacqWGdbWqaeaadaqdaaqaaiabdAgaMbaaaaaacaGLOaGaayzkaaGaeiilaWcaaa@4D15@

where H(C)=∑ip(ci)log⁡p(ci)
 MathType@MTEF@5@5@+=feaafiart1ev1aaatCvAUfKttLearuWrP9MDH5MBPbIqV92AaeXatLxBI9gBaebbnrfifHhDYfgasaacH8akY=wiFfYdH8Gipec8Eeeu0xXdbba9frFj0=OqFfea0dXdd9vqai=hGuQ8kuc9pgc9s8qqaq=dirpe0xb9q8qiLsFr0=vr0=vr0dc8meaabaqaciaacaGaaeqabaqabeGadaaakeaacqWGibasdaqadaqaaiabdoeadbGaayjkaiaawMcaaiabg2da9maaqafabaGaemiCaa3aaeWaaeaacqWGJbWydaWgaaWcbaGaemyAaKgabeaaaOGaayjkaiaawMcaaiGbcYgaSjabc+gaVjabcEgaNbWcbaGaemyAaKgabeqdcqGHris5aOGaemiCaa3aaeWaaeaacqWGJbWydaWgaaWcbaGaemyAaKgabeaaaOGaayjkaiaawMcaaaaa@44AD@ : feature, *c *class

### Logistic scheme

The feature selection method assigns a score to every feature in the feature set, based on the intrinsic properties of the dataset such as feature-class entropy. Our feature construction method for the composite positional features expanded the feature set by adding a new nucleotide from any position in the sequence to the original feature. We added a score that penalizes this distance, such that the farther away the position of the newly added nucleotide to the original feature is, the lower the score of the newly constructed feature. We normalized the distance values to a standard normal distribution. Then we applied a logistic scheme to assign these scores to each of the features in the constructed set of positional features. Finally, each feature was assigned a score according to the following formula:

*F*_*score *_= *IG*(*f*) + exp(*dist*^-1^)

### Feature Generation Algorithm (FGA)

By employing a systematic search over the feature space, we can extract relevant features more efficiently than if a single selection were applied to the whole set. The feature generation algorithm combines feature-construction and feature-selection methods described above, it chooses a final set of features, and it produces a classification model for the task at hand. The algorithm is composed of these main stages:

• *Feature Generation*. We start with an initial set of features. For each iteration we follow these steps: 1) we expand the given features obtaining a new set of composite features during the feature-construction step, and 2) we specify the useful ones during the feature-selection step. The features that are assigned a low selection score by the feature-selection method and logistic scheme are eliminated. We repeat using the selected features as input for the construction method and iterate through these steps to generate richer and more complex features until a specified number of iterations is reached.

• *Final Feature Collection and Selection*. In this stage, we collect all the features selected on each iteration and apply another feature-selection step.

• *Classification*. The last stage of our algorithm builds a classifier over the final set of features. The learned parameters are used to classify new sequences.

While feature generation remains a computationally intensive process, the organization of the generation process according to the different types allows us to search a much larger space of features efficiently. This provides us with a set of different composite features, which may prove valuable for the task at hand. However, when the training is complete the classification stage has a linear complexity, depending only on the number of putative splice-sites present in the input sequence.

## Authors' contributions

All authors participated in the design of the study and the interpretation of results. All authors contributed, read, and approved the final manuscript.

## Supplementary Material

Additional file 1 **FGA identified features that contribute to **Table 2. **(Table2_features_A3mer3.txt)**. A text file with the complete list of features associated with the branch-point interval [-40,-20], from the feature set A-3mer3. The features are ranked according to the absolute value or their assigned weight. The top-scoring 20 features of this list are shown in Table 2. Click here for file

Additional file 2 **Features that contribute to **Figure 2** and other features that show similar behaviour (tetramers-of-figure2.txt). A text file with the complete list of selected features from feature set A-3mer1 [-60,-5].** Click here for file

Additional file 3 **FGA identified hexamers in acceptor splice-site prediction and donor splice-site prediction (FGA-hexamers.txt)**. A text file with the complete list of hexamers that our method indicates they are likely to be ESEs or ESSs. Click here for file

Additional file 4 **FGA-generated features produce a significant overlap with experimentally identified ESE sequences table in **[22]** (ESE-ESS-overlap-sequences.xls)**. The first worksheet in the Excel file contains the table of experimentally identified ESE sequences in [22] and the overlap with the FGA identified hexamers from feature sets A-6mer [0,80] and D-6mer [2,82]. For each comparison an exact match is required. We compared the positively weighted hexamer sets against the ESE sequences, and the negatively weighted hexamer sets against ESS sequences. The second worksheet contains the overlap of the ESE sequences with the FGA identified hexamers that are not included in RescueESE, AstESR or ChPESE sets. Click here for file

Additional file 5 **FGA-generated features produce significant overlap with computationally identified lists of exonic splicing regulator signals **[23,26]** (candidate-ese-esr-overlap.txt)**. A text file with the list of FGA features overlapping with RescueESE and AstESR exonic splicing regulator lists. Click here for file
